# Role of albumin in regulating platelet function

**DOI:** 10.3389/fphar.2026.1734694

**Published:** 2026-02-20

**Authors:** Qiwen Tai, Mengnan Yang, Shuang Chen, Yue Xia, Chenglin Sun, Lili Zhao, Kangxi Zhou, Pixia Gong, Jie Shen, Qiuxia Huang, Qing Li, Renping Hu, Rong Yan, Kesheng Dai

**Affiliations:** 1 Jiangsu Institute of Hematology, the First Affiliated Hospital of Soochow University, NHC Key Laboratory of Thrombosis and Hemostasis, National Clinical Research Center for Hematologic, Suzhou, China; 2 Department of Pathology and Pathophysiology and Bone Marrow, Transplantation Center of the First Affiliated Hospital, Zhejiang University School of Medicine, Hangzhou, China

**Keywords:** albumin, hypoproteinemia, platelet activation, signal transduction pathway, thrombus

## Abstract

**Introduction:**

Hypoproteinemia, which occurs in diverse clinical conditions, can cause a range of complications such as thrombosis, which is among the most serious and potentially life-threatening. Albumin is a widely utilized clinical therapeutic agent; however, research regarding its regulatory effects on thrombosis remains limited, and existing clinical evidence presents conflicting findings. The precise mechanisms whereby albumin affects platelet thrombus formation require further investigation.

**Methods:**

After co-incubating albumin with platelets, various platelet function experiments were carried out. The participation of signaling pathways and protein structures in the mechanism was verified by means of Western blot technology, protein charge neutralization, and over expression of molecules. Validation was also conducted through the construction of animal models.

**Results:**

Albumin significantly inhibited platelet thrombus formation in a murine model of hypoproteinemia without inducing hemorrhagic risk. Platelet aggregation, integrin activation on the membrane surface, and ATP release induced by various agonists were all inhibited. Transmission electron microscopy and fluorescence confocal microscopy revealed that albumin could suppress granule release, alter granule distribution within platelets, and inhibit platelet spreading. Furthermore, albumin was found to reduce the phosphorylation levels of PKC and Akt in the platelet activation signaling pathway. By neutralizing the surface negative charge of albumin and adding cationic surfactants such as quaternary ammonium salts, we confirmed that the surface negative charge of albumin was critical to the inhibition of platelet aggregation and granule release.

**Conclusion:**

Albumin can inhibit platelet activation and thrombus formation through the negatively charged surface residues and by modulating the PKC and Akt signaling pathways.

## 1 Introduction

Characterized by abnormally low levels of protein in the blood, hypoproteinemia is an independent risk factor for adverse outcomes in critically ill patients. It can lead to various complications, including thrombosis, which is one of the most serious and potentially life-threatening (Valeriani et al., 2024). Hypoproteinemia frequently occurs in conditions such as liver cirrhosis, nephrotic syndrome, and severe burns (Wei et al., 2024; Misirocchi et al., 2025; Abedi et al., 2024). Clinical evidence reveals altered coagulation profiles in patients with nephrogenic hypoproteinemia and a significant negative correlation between plasma albumin levels and the severity of thrombosis, underscoring a potential mechanistic link (Zuin and Rigatelli, 2024; Wu et al., 2023). However, the pathophysiological mechanisms connecting hypoproteinemia to heightened thrombotic risk are complex and remain poorly defined, likely involving multiple regulatory networks (Zheng et al., 2024; Song et al., 2025; Das et al., 2024).

As the most abundant protein in human plasma, albumin concentrations usually range from 35.0 to 55.0 g/L, which is approximately 50% of total plasma protein (Abedi et al., 2024). The albumin structure typically contains a single polypeptide chain consisting of 585 amino acid residues arranged in an oval shape (Sleep, 2015). Albumin serves as the primary natural colloid molecule in the human body and has a critical role in maintaining nutritional balance and osmotic pressure stability (Li et al., 2025; Wiedermann, 2022). Beyond these classical roles, emerging clinical observations suggest that albumin supplementation may improve patient outcomes and serve as an adjunct in preventive anticoagulation therapy (Marcos-Contreras et al., 2023; Uno et al., 2024). This hints at a direct, modulatory role for albumin in hemostasis and thrombosis, potentially through the stabilization of vascular endothelial function (Iba et al., 2016). Despite its clinical relevance, the direct impact of albumin on platelet function, which is a key cellular mediator of thrombosis, remains controversial.

We conducted a review of the previous studies and identified unresolved contradictions concerning the effect of albumin on platelets. Early researchers employed nagase analbuminemic rats (NARs) and human platelets in their investigations and ascertained that albumin could augment collagen - induced platelet aggregation (Sekiya et al., 1988; Sekiya et al., 1990). Conversely, other studies demonstrated that albumin could impede platelet aggregation initiated by other agonists, including platelet - activating factor (PAF), thrombin, and histones (Jorgensen and Stoffersen, 1980; Grigoriadis and Stewart, 1992; Sloand et al., 1992; Lam et al., 2013). This evident disparity in research findings implies that there are substantial lacunae in our comprehension of the role of albumin in platelet cell biology. Subsequent exploration of the mechanism was limited and inconclusive, ascribing the effect to variables such as alterations in fatty acid binding or phosphatidylinositol metabolism, yet failing to offer a unified explanation (Imada et al., 1981; Amin et al., 1991; Kirman et al., 1999). As a result, the interaction between native albumin and platelets remains poorly explored.

Critically, prior studies have been constrained by several crucial factors: (1) Platelet aggregation experiments were employed as the sole endpoint of the research, overlooking other significant aspects of platelet activation, including integrin activation, P-selectin release, platelet granule secretion, and procoagulant activity; (2) There was a lack of research on the possible regulation of intracellular signaling pathways in platelets by albumin; (3) In the thrombosis environment, no controlled model was utilized for direct *in vivo* validation to distinguish the effects of albumin deficiency from other confounding variables. To resolve these longstanding inconsistencies and definitively characterize the direct role of albumin in platelet regulation and thrombus formation, we designed a comprehensive study. We hypothesized that physiological and pathological concentrations of albumin exert a broad inhibitory effect on platelet function by modulating key intracellular signaling hubs, specifically the protein kinase C (PKC) and Akt pathways, and that this effect is intrinsically linked to its biophysical properties.

To test this hypothesis, we employed an integrated, multi-level strategy: (1) systematic *in vitro* analysis of washed human platelets exposed to a clinically relevant concentration range of albumin, assessing a comprehensive panel of functional responses (aggregation, integrin αIIbβ3 activation, granular secretion, and spreading) to various agonists; (2) mechanistic dissection of PKC/Akt signaling and the specific role of albumin’s surface charge; and (3) development and utilization of a novel murine model of adriamycin-induced acute kidney injury accompanied by hypoproteinemia to evaluate platelet hyperactivity and thrombus formation *in vivo*, and the therapeutic potential of albumin replenishment. This approach aims to provide conclusive evidence, clarifying albumin’s direct effects on platelets and establishing its potential as an endogenous modulator of thrombosis.

## 2 Materials and methods

### 2.1 Human studies

Healthy adult volunteers were recruited for median cubital vein blood collection. These individuals had abstained from any antiplatelet medications for at least 2 weeks prior to donation and reported adequate rest. All blood samples collected were exclusively for scientific research purposes, with informed consent obtained in advance. The study was approved by the Medical Ethics Committee of the First Affiliated Hospital of Soochow University.

### 2.2 Animals

The BALB/c wild-type (WT) mice were procured from Changzhou Laboratory Animal Co., Ltd. (China). Soochow University provides specific pathogen free (SPF) grade animal housing facilities for laboratory animals. The experimental animals utilized in this study were housed in the SPF grade facility at Soochow University, and all animal experiments were conducted in strict compliance with the guidelines of animal ethics, utilizing pentobarbital sodium or isoflurane gas anesthesia for both experimentation and euthanasia. These procedures have been approved by the Medical Ethics Committee of the First Affiliated Hospital of Soochow University. Male BALB/c mice, weighing approximately 20 g and aged around 6 weeks, were selected for the study. The ADR mouse model was established by administering a single intravenous injection of doxorubicin hydrochloride at a dose of 7.5 mg/kg via the tail vein. Doxorubicin hydrochloride was dissolved in 0.9% sodium chloride solution. Two weeks post-injection, kidney histopathological staining and plasma protein concentration were evaluated to confirm successful model establishment. The ADR Albumin mouse model was further developed by injecting mouse albumin at a dose of 30 mg once a week through the intraorbital venous plexus, repeated twice.

### 2.3 Reagents

Human plasma albumin (70024-90-7), thrombin (10602-40-001), U46619 (5685-40-1), ADP (58-64-0), fibrinogen (9001-32-5), glycine methyl ester hydrochloride (5680-79-5), N-(3-dimethylaminopropyl)-N′-ethylcarbodiimide hydrochloride (EDC) (25952-53-8), and doxorubicin hydrochloride (25316-40-9) were obtained from Sigma-Aldrich (St. Louis, MO, United States). FITC-labeled anti-human P-selectin (clone AK4, 304904) was purchased from BioLegend Corporation (San Diego, CA, United States). FITC-labeled anti-human PAC-1 (340507) was sourced from BD Biosciences (San Jose, CA, United States). FITC-conjugated rat anti-mouse P-selectin (clone Wug.E9, M130-1) and PE-conjugated rat anti-mouse Integrin αIIbβ3 (M023-2) were obtained from Emfret Analytics (Eibelstadt, Germany). Collagen (385) and Chrono-Lume (395) were provided by Chrono-Log Corporation (Havertown, PA, United States). PKC activator PMA (HY-18739), PKC activator PDBu (HY-18985), Akt activator 740Y-P (HY-P0175), and Akt activator SC79 (HY-18749) were acquired from MedChemExpress (Monmouth Junction, NJ, United States). Mouse serum albumin (SEKM-0297), Tris-Base, bovine serum albumin (BSA), tissue fixative solution, loading buffer solution, and glycine were purchased from suppliers in Beijing, China. Alexa Fluor 488-phalloidin (A12379) and the Slip-Alyzer dialysis kit with a 20 kDa molecular weight cut-off (MWCO) (66003) were obtained from Thermo Fisher Scientific (Rockford, IL, United States). An SDS-PAGE adhesive kit (10%) was purchased from Epizyme Biotech Co., Ltd. (Shanghai, China). Rabbit antibodies against phospho-PKC substrate (2261), phospho-Akt (Ser473) (11E7), Akt (pan) (11E7), PKC alpha (2056), GAPDH (5174), cell lysate buffer (9803), and PAR4-AP (2328) were sourced from Cell Signaling Technology, Inc. (Beverly, MA, United States). HRP-conjugated goat anti-mouse IgG (A0216), anti-rat IgG (A0192), and anti-rabbit IgG (A0208) were obtained from Beyotime Biotechnology (Shanghai, China). Mouse albumin, prealbumin, total protein, and globulin ELISA testing kits were procured from Aifang Biotechnology Co., Ltd. (Hunan, China). The biochemical reagents utilized for the preparation of modified tyrode’s buffer (MTB) and acid-citrate-dextrose (ACD) blood preservative solutions included citric acid, glucose, sodium citrate, sodium chloride, magnesium chloride, and EDTA. Magnesium chloride and EDTA were sourced from suppliers in Suzhou, China.

### 2.4 Platelet preparation

Male WT, ADR, and ADR Albumin mice weighing 20∼25 g and approximately 8 weeks old were selected for the experiment. Blood was collected using ACD anticoagulant at a ratio of 1:7 (ACD: whole blood). The ACD solution contained 2.5% trisodium citrate, 2.0% D-glucose, and 1.5% citric acid. The mixture was centrifuged at 1,100 rpm for 8 min to separate platelet-rich plasma (PRP). PRP was then subjected to a second centrifugation at 3,500 rpm for 2 min. The supernatant was discarded, and platelets were washed with CGS buffer. Following another centrifugation at 2,500 rpm for 2 min, the supernatant was discarded, and the washing process was repeated with 1 mL of CGS buffer. After a final centrifugation the supernatant was removed, and the platelets were resuspended in MTB buffer. 1 M CaCl_2_ and 1 M MgCl_2_ were added at a ratio of 1:1000.

### 2.5 Flow cytometry

Following the selection of resting platelets, thrombin, U46619, PAR4-AP, and Trap-6 were introduced as platelet agonists and incubated at 37 °C for 30 min. Subsequently, 10 μL of the treated platelets were transferred to a flow cytometry tube, followed by the addition of 1.5 μL of fluorochrome-conjugated monoclonal antibody. The mixture was then incubated in the dark for 15 min. To terminate the reaction, 350 μL of MTB was added, and flow cytometry analysis was performed as soon as possible.

### 2.6 Aggregation

The platelet aggregometer was preheated to 37 °C prior to the experiment. A volume of 250 μL of washed platelets was introduced into an aggregation tube, followed by the addition of various platelet agonists including thrombin, U46619, collagen, PAR4-AP, Trap-6, and ADP. The platelet aggregation rates elicited by these different agonists were recorded at a stirring speed of 1,200 rpm.

### 2.7 ATP release

A total of 250 μL of washed platelets were introduced into the aggregometer tube. Lume and ATP standard solution were sequentially added to achieve a maximum ATP release value between 40% and 80%. The volumes of lume added and the corresponding ATP release percentages were meticulously recorded. Following this step, various agonists including thrombin, U46619, collagen, PAR4-AP, Trap-6, and ADP were selected for further analysis.

### 2.8 Platelet spreading

A slide coated with 30 μg/mL fibrinogen was utilized. Blocking was performed using 5% BSA at room temperature for 2 h. Rested platelets at a concentration of 2 × 10^7^/mL were stimulated with platelet agonists and promptly transferred to the slide at approximately 20 μL per well, spreading at 37 °C for 40 min. The slide was then washed three times and fixed with 4% PFA for 10 min. Permeabilization was achieved using 0.1% Triton-X100. Blocking was repeated with 5% BSA for 1 h. F-actin in the platelets was labeled with Alexa Fluor 488-phalloidin in a light-protected environment. Platelet spreading was observed under a Leica confocal microscope using a 63 × oil immersion lens.

### 2.9 FeCl_3_ induced mesenteric artery thrombosis model

WT, ADR, and ADR Albumin mice weighing 20∼25 g and approximately 8 weeks old were selected for intraperitoneal injection of 1% pentobarbital sodium for anesthesia. The fluorescent indicator calcein was added at a ratio of 1:300, and platelets were incubated in the dark for 30 min. Four-week-old WT mice underwent gas anesthesia followed by light anesthesia, after which calcein-labeled washed platelets were transfused via the medial orbital canthus venous plexus. Recipient mice were placed on their side on a clear glass plate and a midline abdominal incision was made. An appropriate arterial vessel was selected, and a 1 mm^2^ filter paper soaked in 6% FeCl_3_ was placed on the vessel. A fluorescence microscope was used to observe thrombus formation at the FeCl_3_ induced injury site, and the time of thrombus formation was recorded.

### 2.10 Tail bleeding time

The mice were positioned horizontally on the operating table. The distal 5 mm of the mouse tail was transected perpendicular to the workbench, and the tail was gently submerged in the preheated normal saline. The bleeding time from the tail was meticulously recorded.

### 2.11 ELISA

The standard substances in the ELISA test kit were serially diluted to prepare the standard curve. Standard substances with gradient concentrations were added to the 96-well plate respectively. Each standard substance should be set up in duplicate or triplicate and gently mixed to avoid cross-contamination. Measured the OD value within 15 min after adding the stop solution using an enzyme-linked immunosorbent assay reader. The standard curve was used to calculate the final concentration values.

### 2.12 Western blot

Added EDTA to the mixture of platelets and albumin at a ratio of 1:500, the supernatant was removed by centrifugation at 2,500 rpm for 2 min, after which the cells were lysed using 1 × cell lysis buffer. Subsequently, 4 × protein loading buffer was added to the lysed platelets at a volume ratio of 1:4. After thorough mixing by vortexing, the samples were heated in a metal bath at 100 °C for 5 min and then stored at −80 °C after cooling to room temperature.

### 2.13 Neutralization of the charge on the albumin surface

A total of 300 mg albumin were dissolved in 2.25 mL of 0.133 M glycine methyl ester hydrochloride solution (pH 4.75). Subsequently, 750 μL of 0.04 M EDC solution (pH 4.75) was added to the albumin solution. Over the following 30 min, 1 mL aliquots of the reaction mixture were withdrawn every 5 min and immediately quenched with 1 mL of 4 M acetate buffer (pH 4.75) to halt the reaction. The resulting solution was then incubated at room temperature for an additional 30 min. Following this, the solution was transferred to a dialysis cassette and subjected to dialysis against 1 L of PBS at room temperature for 2 h under gentle stirring conditions. The dialysis was subsequently continued overnight at 4 °C. After dialysis, the solution was removed from the cassette and concentrated using an ultrafiltration tube. The isoelectric point of the protein was determined by two-dimensional electrophoresis, and its secondary structure was characterized by circular dichroism spectroscopy.

### 2.14 Statistical analysis

Statistical analysis of the experimental data was conducted using GraphPad Prism 9.0 software. Differences between groups were assessed by one-way ANOVA and followed by Dunnett’s *post hoc* test. Statistics are presented as mean ± SEM. *P < 0.05, **P < 0.01 and ***P < 0.001. The two-tailed Student’s t-test was utilized to compare data between two groups. Each experiment was replicated at least three times. All animal experiments were randomized, and no animals or samples were excluded from the study or data analysis.

## 3 Results

### 3.1 Human plasma albumin exhibited inhibitory effects on platelet aggregation, integrin αIIbβ3 activation and P-selectin release

**FIGURE 1:**
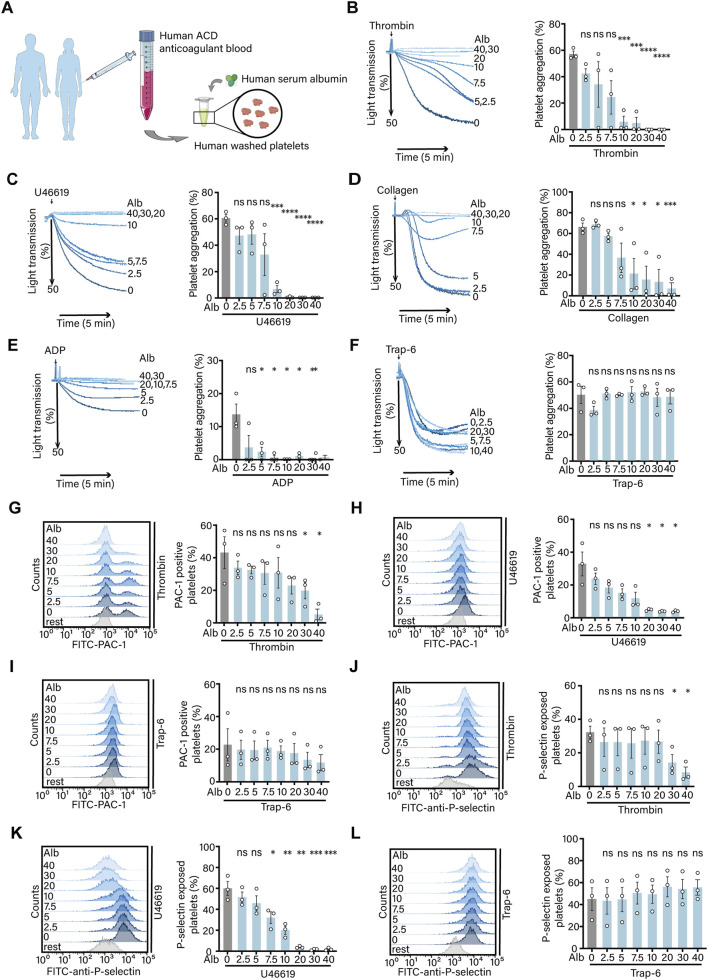
Human plasma albumin exhibits inhibitory effects on platelet aggregation, integrin αIIbβ3 activation and P-selectin release. **(A)** Schematic diagram of the experimental procedure. **(B–F)** Effect of human plasma albumin (PBS, 2.5, 5.0, 7.5, 10.0, 20.0, 30.0, 40.0 mg/mL) on 0.01 U/mL thrombin **(B)**, 200 nM U46619 **(C)**, 1 μg/mL collagen **(D)**, 200 μM ADP **(E)**, 20 μM Trap-6 **(F)** induced aggregation of human washed platelets (n = 3 independent experimental subjects). **(G–I)** Effect of different concentrations of human plasma albumin on thrombin **(G)**, U46619 **(H)** and Trap-6 **(I)** induced activation of human washed platelet integrin αIIbβ3 (n = 3 independent experimental subjects). **(J–L)** Effect of human plasma albumin on thrombin **(J)**, U46619 **(K)** and Trap-6 **(L)** induced release of P-selectin from human washed platelets (n = 3 independent experimental subjects). Differences between groups were assessed by one-way ANOVA followed by Dunnett’s *post hoc* test (compared with PBS). Statistics are presented as mean ± SEM. *P < 0.05, **P < 0.01 and ***P < 0.001.

In clinical medicine, it is observed that patients with portal vein thrombosis exhibit lower plasma albumin levels compared to healthy controls (Zhou and Fang, 2020). To investigate the effect of albumin on platelet activation, we incubated human washed platelets with different concentrations of albumin, then stimulated platelets with agonists and detected the effect of albumin on platelet activation (Figure 1A). The results showed that, albumin significantly inhibited platelet aggregation induced by thrombin (Figure 1B), U46619 (Figure 1C), collagen (Figure 1D) and ADP (Figure 1E). However, albumin had no effect on platelet aggregation induced by Trap-6 (Figure 1F). Based on the results from the platelet aggregation experiment, we selected thrombin and U46619, which exhibited significant inhibitory effects, as well as Trap-6 with no inhibitory effect, for further experimentation. Both thrombin and U46619-induced platelet integrin αIIbβ3 activation were inhibited by albumin in a concentration-dependent manner (Figures 1G,H). However, consistent with the results of platelet aggregation, platelet integrin αIIbβ3 activation induced by Trap-6 also showed no significant differences at each albumin concentration (Figure 1I). Upon platelet activation, P-selectin is rapidly translocated to the platelet surface (Yan et al., 2024). Therefore, we subsequently investigated the release of P-selectin. The results showed that albumin significantly inhibited thrombin (Figure 1J) and U46619 (Figure 1K) induced P-selectin release from human washed platelets. However, Trap-6 induced P-selectin release did not show significant differences in each group (Figure 1L).

### 3.2 Albumin inhibited granule release and spread of platelet

**FIGURE 2:**
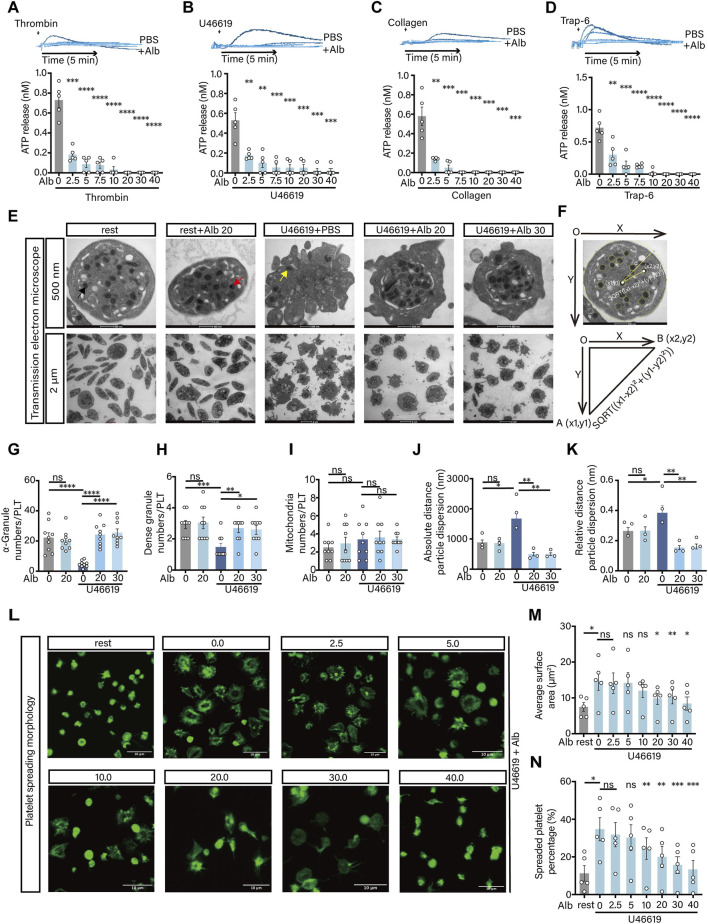
Albumin inhibited granule release and spread of platelet. **(A–D)** Effect of human plasma albumin on 0.02 U/mL thrombin **(A)**, 600 nM U46619 **(B)**, 3 μg/mL collagen **(C)** and 40 μM Trap-6 **(D)** induced effects on human washed platelet ATP release (n = 5 independent experimental subjects). **(E–K)** Transmission electron microscopy field of view of platelets induced by 200 nM U46619 at different concentrations of human plasma albumin (20, 30 mg/mL) **(E)**, platelet cell diameters using ImageJ fitting graphs and axes to find the distance of granules from the centre of the cell **(F)**, absolute counts of α-granules **(G)**, absolute counts of dense granules **(H)**, absolute counts of mitochondria **(I)**, absolute distance of granules from the centre of the cell **(J)**, relative distance of granules from the centre of the cell **(K)** (**E–I**, n = 3 independent experimental subjects, counted 3 microscope field of view per individual; **J,K**, n = 3 independent experimental subjects, counted the distance from all granules to the center of the cell and calculated the average). **(L–N)** Inhibitory effect of human plasma albumin on U46619 induced platelet spreading **(L)**, mean spreading area of a single platelet **(M)**, spreading platelets as a percentage of total platelets in the field of view **(N)** (n = 5 independent experimental subjects). Differences between groups were assessed by one-way ANOVA followed by Dunnett’s *post hoc* test (compared with PBS in **A–G**). Statistics are presented as mean ± SEM. *P < 0.05, **P < 0.01 and ***P < 0.001.

When platelets are stimulated, granules can fuse with the platelet open canalicular system (OCS) and be released into the extracellular environment (Chung et al., 2021). We then investigated the effect of albumin on platelet ATP release. Surprisingly, at the concentrations up to 2.5 mg/mL, albumin could significantly inhibited platelet ATP release induced by all agonists, including Trap-6 (Figures 2A–D). The above results indicated that albumin could significantly inhibit platelet granule release. We then used transmission electron microscopy to observe the distribution of granules in platelets (Figures 2E,F). The results showed that resting platelets had rounded edges and contained a large number of granules and OCS in their interior. Activated platelets, on the other hand, formed a large number of pseudopods that aggregated together. Compared to resting platelets, the numbers of α-granules and dense granules were significantly reduced in activated platelets (Figures 2G,H), whereas the number of mitochondria was not different (Figure 2I). Surprisingly, when we incubated albumin with platelets for 15 min before stimulated the platelets with agonists, the number of α-granules and dense granules inside the platelets increased significantly (Figures 2G,H), which was consistent with our observation that albumin had a strong inhibitory effect on the release of ATP from platelets. We also counted the percentages of α-granules (Supplementary Figure S1A), dense granules (Supplementary Figure S1B) and mitochondria (Supplementary Figure S1C), the results were consistent with the statistics of their absolute counts. By morphological observation, we found that platelets incubated with albumin had fewer pseudopods and a reduced spreading area in the 500 nm field of view. Interestingly, by measuring the absolute and relative distance (Figures 2J,K) of the granules from the center of the platelet, we also found that after incubation with albumin, the granules of activated platelets are crowded in the central region of the cell surrounded by the OCS. In the 2 μm field of view, the aggregation of activated platelets incubated with albumin was significantly improved. Stimulation of second-phase platelet aggregation by U46619 requires integrin outside-in signaling (Yan et al., 2024). To investigate whether albumin is involved in this signaling pathway, the effect of albumin on platelet spreading was examined. The results demonstrated that, albumin inhibited platelet spreading in a concentration-dependent manner after stimulation with U46619 (Figure 2L). By marking the cytoskeletal proteins of platelets, it was found that albumin could suppress the spreading area of activated platelets (Figure 2M) and the proportion of spread platelets (Figure 2N) in a concentration-dependent manner.

### 3.3 Albumin inhibited platelet activation by inhibiting PKC and Akt phosphorylation

**FIGURE 3:**
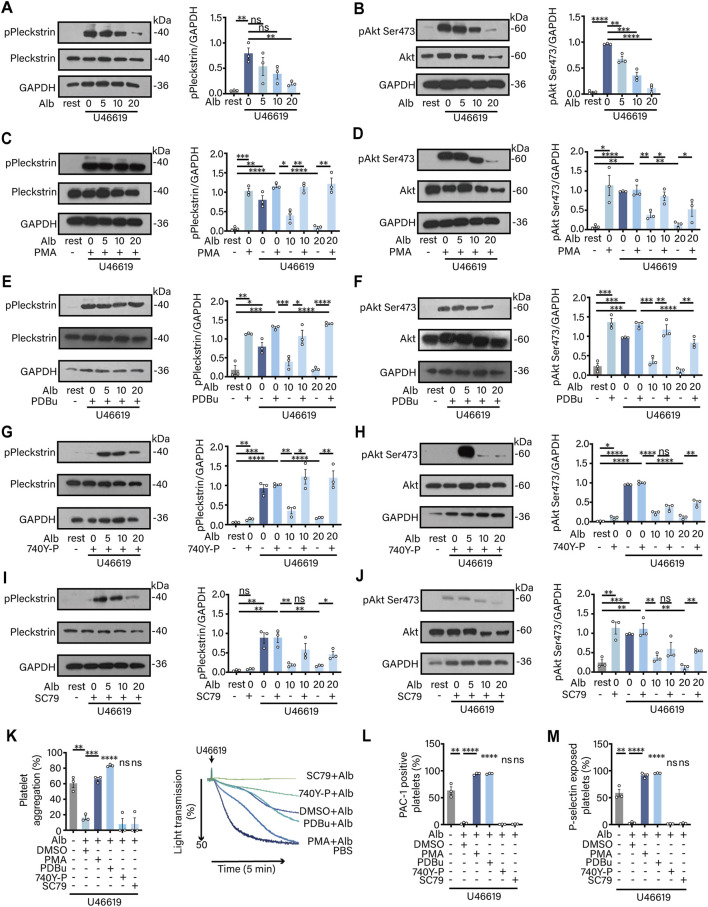
Albumin inhibited platelet activation by inhibiting PKC and Akt phosphorylation. **(A,B)** Effect of human plasma albumin (5.0, 10.0 and 20.0 mg/mL) on pleckstrin phosphorylation **(A)** and Akt Ser473 phosphorylated **(B)** expression in human washed platelets induced by U46619. **(C,D)** Effect of human plasma albumin and 2 nM PMA on the expression of pleckstrin phosphorylation **(C)** and Akt Ser473 phosphorylation **(D)** in human washed platelets induced by U46619. **(E,F)** Effect of human plasma albumin and 20 nM PDBu on pleckstrin phosphorylation **(E)** and Akt Ser473 phosphorylation **(F)** expression induced by U46619 in human washed platelets. **(G,H)** Effect of human plasma albumin and 20 nM 740Y-P on pleckstrin phosphorylation **(G)** and Akt Ser473 phosphorylation **(H)** expression in human washed platelets induced by U46619. **(I,J)** Effect of human plasma albumin and 8 μg/mL SC79 on pleckstrin phosphorylation **(I)** and Akt Ser473 phosphorylation **(J)** expression in human washed platelets induced by U46619 (**A–J**, n = 3 independent experimental subjects). **(K–M)** Effect of 20.0 mg/mL concentration of human plasma albumin and PMA, PDBu, 740Y-P and SC79 on U46619-induced aggregation **(K)**, integrin αIIbβ3 activation **(L)** and P-selectin release **(M)** in human washed platelets (n = 5 independent experimental subjects). Differences between groups were assessed by one-way ANOVA followed by Dunnett’s *post hoc* test. Statistics are presented as mean ± SEM. *P < 0.05, **P < 0.01 and ***P < 0.001.

Cyclic guanosine monophosphate (cGMP) regulates various signaling pathways that inhibit platelet activation, albumin can indirectly regulate cGMP by regulating the duration of NO (Gansevoort et al., 2025). Consequently, this study aims to investigate whether albumin has a direct effect on platelet signaling. Members of the PKC family serve as crucial effector molecules for second messengers such as calcium ions and diacylglycerol, while Akt functions as a serine and threonine kinase (Zhang et al., 2024; Sun et al., 2023). To assess whether albumin can directly regulate PKC and Akt phosphorylation, we activated platelets after co-incubation with albumin and observed the phosphorylation levels of PKC and Akt. The results showed that albumin was able to significantly inhibit U46619 induced phosphorylation of PKC and Akt in a dose-dependent manner (Figures 3A,B). When we used PMA, an activator of PKC, the inhibitory effect of albumin on PKC and Akt phosphorylation was restored (Figures 3C,D). The same phenomenon was observed when we used PDBu, the other activator of PKC (Figures 3E,F). The inhibitory effect of albumin on PKC and Akt phosphorylation was similarly restored when we used 740Y-P, the activator of Akt (Figures 3G,H), but not as dramatically as with the PKC activator. When the other Akt activator, SC79 was used, the results were not different from those obtained with 740Y-P (Figures 3I,J). The aforementioned findings demonstrated that albumin exerted a significant inhibitory effect on PKC and Akt phosphorylation. Furthermore, it appeared that PKC and Akt did not exhibit a clear upstream-downstream relationship in the context of albumin’s inhibition of platelet activation. Subsequently, we incubated platelets with 20 mg/mL albumin, followed by treatment with four kinase activators, and stimulated the platelets with U46619. We then measured platelet aggregation, P-selectin release, and integrin αIIbβ3 activation levels. The results indicated that PKC activators, PMA and PDBu, could significantly reversed the inhibitory effects of albumin on platelet aggregation, P-selectin release, and integrin αIIbβ3 activation. In contrast, Akt activators, 740Y-P and SC79, failed to restore these inhibitory effects (Figures 3K–M).

### 3.4 Albumin influenced platelet aggregation, ATP release, granule release and internal granule arrangement via surface charge

**FIGURE 4:**
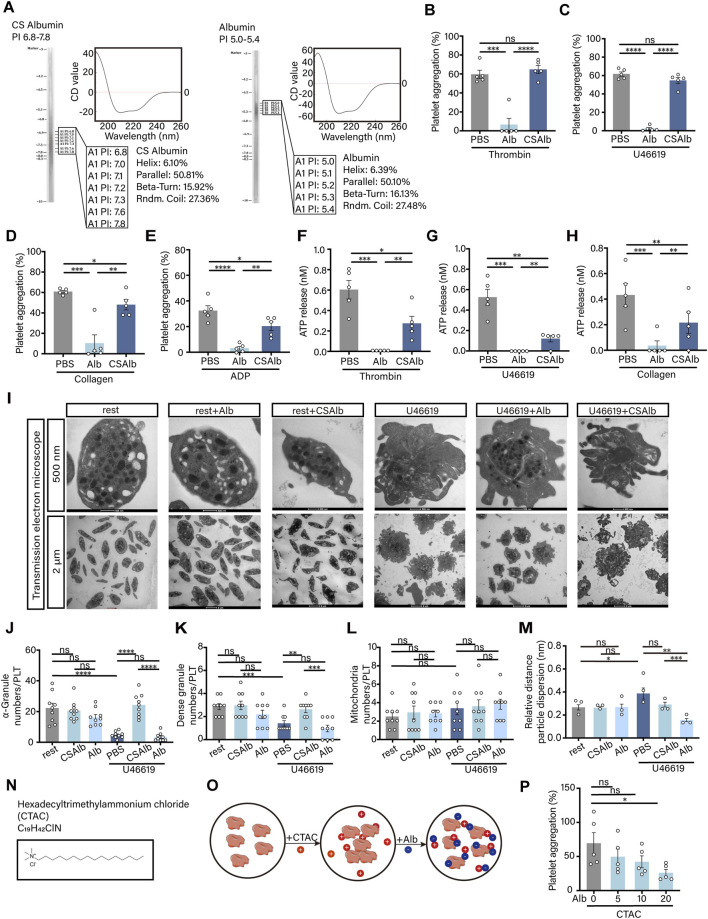
Albumin influenced platelet aggregation, ATP release, granule release and internal granule arrangement via surface charge. **(A)** Isoelectric point detection of CS albumin and natural albumin (80 mg/mL) and determination of protein secondary structure by circular dichroism. **(B–E)** Effect of CS albumin and natural albumin (20.0 mg/mL) on human washed platelet aggregation induced by thrombin **(B)**, U46619 **(C)**, collagen **(D)** and ADP **(E)**. **(F–H)** Effect of CS albumin and natural albuminon ATP release from human washed platelets induced by 0.02 U/mL thrombin **(F)**, 500 nM U46619 **(G)** and 3 μg/mL collagen **(H)** (**B–H**, n = 5 independent experimental subjects). **(I)** Transmission electron microscopy was used to observe the effect of CS albumin and natural albumin on the internal granule arrangement in platelets before and after activation induced by U46619. **(J–M)** Effects of CS albumin and natural albumin on the absolute number of α-granules **(J)**, dense granules **(K)**, mitochondria **(L)** and the relative distance of granules from the centre of the cell **(M)** in platelets induced by U46619 (**J–L**, n = 3 independent experimental subjects, counted 3 microscope field of view per individual; **(M)** n = 3 independent experimental subjects, counted the distance from all granules to the center of the cell and calculated the average). **(N)** Molecular structure of cationic surface activator, quaternary ammonium salt. **(O)** Flowchart of experiments using albumin to inhibit platelet aggregation responses induced by CTAC. **(P)** Inhibitory effects of different concentrations of albumin (5, 10 and 20 mg/mL) on 50 nM CTAC-induced platelet aggregation (n = 5 independent experimental subjects). Differences between groups were assessed by one-way ANOVA followed by Dunnett’s *post hoc* test. Statistics are presented as mean ± SEM. *P < 0.05, **P < 0.01 and ***P < 0.001.

In the peripheral blood environment, albumin typically exhibits a negative surface charge (Sleep, 2015). Literature also indicates that the internal granules of platelets carry a negative charge (Chung et al., 2021). Based on these background studies and our previous observations, we hypothesized that albumin could suppress platelet activation via its charge properties. We observed that adjusting the pH of the solvent equalized the acidic and alkaline dissociations of the albumin molecules, thereby neutralizing the positive and negative surface charges (Supplementary Figure S2). Consequently, the isoelectric point shifted from 5.0 to 5.4 to 6.8∼7.8 (Figure 4A). Additionally, we analyzed the secondary structure of the neutralized albumin using circular dichroism spectroscopy and found that the structures remained unchanged compared to those of native albumin. These findings indicated that the neutralization of surface charges was achieved without compromising the secondary structure of the albumin. We then tested the effect of charge stripped albumin (CS Albumin) on platelet aggregation and ATP release. The results showed that the inhibitory effect of albumin on platelet aggregation induced by thrombin (Figure 4B), U46619 (Figure 4C), collagen (Figure 4D) and ADP (Figure 4E) could be all reversed by neutralising the surface charge. The inhibitory effects of albumin on platelet ATP release were also reversed (Figures 4F–H). Subsequently, the release and arrangement of platelet granules were examined using transmission electron microscopy (Figure 4I). The results showed that there was no significant difference in the number of platelet granules in the resting state after co-incubation with albumin or CS albumin. However, when platelet activation was induced, the effect of albumin on the number of platelet granules was reversed by charge neutralisation (Figures 4J,K). The absolute counts of mitochondria were not significantly different in any of the groups (Figure 4L). The relative count statistics for the three organelles were consistent with the absolute count results (Supplementary Figures S3A–C). The effect of albumin on the degree of platelet granule discrete was also restored with both the relative distance (Figure 4M) and the absolute distance (Supplementary Figure S3D) of granules from the platelet center. Next, we used a cationic surface activator, a type of quaternary ammonium salt, hexadecyltrimethylammonium chloride (CTAC) for validation (Figure 4N). We directly induced the platelet aggregation with CTAC without using any platelet agonist and determined whether albumin could directly inhibit platelet aggregation induced by the cationic surface activator (Figure 4O). The results demonstrated that the CTAC induced platelet aggregation was progressively inhibited as the albumin concentration increased (Figure 4P). These findings suggested that the surface charge of albumin exerted a significant influence on inhibiting platelet aggregation, suppressing ATP release, preventing granules release, and altering the internal structural organization of platelets.

### 3.5 Development and characterization of a murine hypoproteinemia model

**FIGURE 5:**
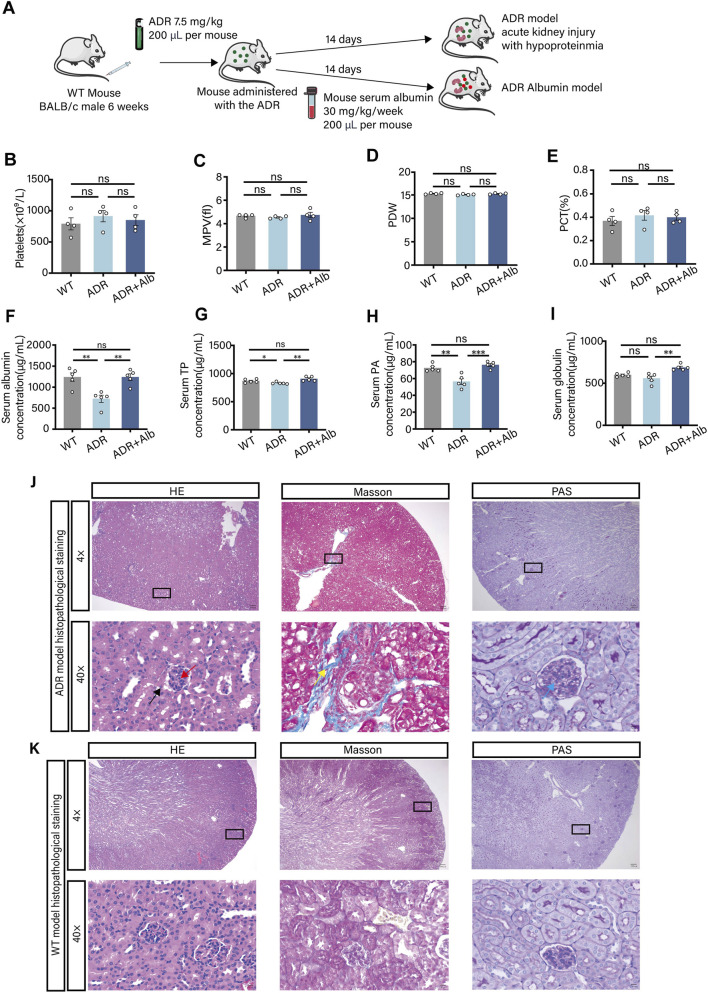
Development of a murine model of hypoproteinemia induced by adriamycin-associated acute kidney injury. **(A)** Flow chart of mouse model construction. **(B–E)** Platelet count **(B)**, MPV **(C)**, PDW **(D)** and PCT **(E)** in peripheral blood of 8-week male WT, ADR and ADR Albumin mice. **(F–I)** Albumin concentration **(F)**, total protein concentration **(G)**, prealbumin concentration **(H)**, globulin concentration **(I)** in peripheral blood of mice (**B–I**, n = 5 independent experimental animals). **(J)** Histopathological staining of kidneys from ADR model mice (4× and 40× magnification), HE staining, Masson staining and PAS staining. **(K)** Histopathological staining of kidney from WT mice (4× and 40× magnification), HE staining, Masson staining, PAS staining. Differences between groups were assessed by one-way ANOVA followed by Dunnett’s *post hoc* test. Statistics are presented as mean ± SEM. *P < 0.05, **P < 0.01 and ***P < 0.001.

Following the *in vitro* demonstration of albumin’s inhibitory effects, we sought to validate these findings *in vivo* using a hypoproteinemia model. We employed the well-established adriamycin (ADR)-induced acute kidney injury model in BALB/c mice, which reliably induces hypoproteinemia with low mortality and high reproducibility (Li et al., 2020; Yu et al., 2024; Cha et al., 2024). We established a renal hypoproteinemia mouse model by administering adriamycin hydrochloride to BALB/c mice and supplemented the model mice with albumin to create a treatment model for hypoproteinemia (Figure 5A). The results demonstrated that there were no significant differences in platelet count, mean platelet volume (MPV), platelet distribution width (PDW), and plateletcrit (PCT) among WT, ADR, and ADR Albumin mice (Figures 5B–E). ELISA analysis of plasma protein concentrations revealed that compared to WT and ADR Albumin mice, the concentration of plasma albumin in ADR mice was significantly reduced (Figure 5F), while the total plasma protein (TP) level remained unaffected (Figure 5G). Notably, the concentration of plasma prealbumin (PA) was markedly decreased in ADR mice (Figure 5H). Additionally, the globulin levels in ADR Albumin mice were slightly elevated compared to those in WT and ADR mice (Figure 5I). Histological examination of mouse kidney tissues using HE, Masson, and PAS staining indicated that, relative to WT mice, the kidneys of ADR mice exhibited moderate glomerular sclerosis, mesenchymal fibrosis, thickening of the glomerular basement membrane, and renal matrix hyperplasia. Cellular crescent formation was also observed within the glomerular sac lumen (Figures 5J,K). In conclusion, the mouse model of adriamycin-induced acute kidney injury leading to hypoproteinaemia had been successfully established.

### 3.6 Hypoproteinemia elevated specific biomarkers of *in vivo* platelet activation

**FIGURE 6:**
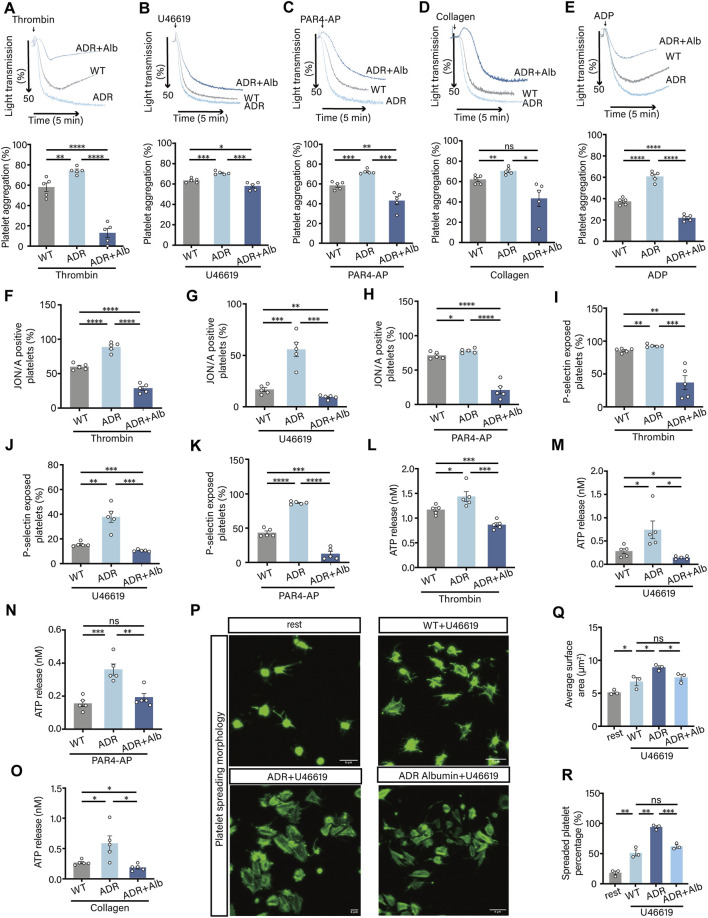
Hypoproteinemia was found to promote platelet activation. **(A–E)** Wash platelet aggregation in WT, ADR and ADR Albumin mice induced by thrombin **(A)**, U46619 **(B)**, PAR4-AP **(C)**, collagen **(D)** and ADP **(E)** (n = 5 independent experimental animals). **(F–H)** Thrombin **(F)**, U46619 **(G)** and PAR4-AP **(H)** induced integrin αIIbβ3 activation in washed platelets from mice (n = 5 independent experimental animals). **(I–K)** Thrombin **(I)**, U46619 **(J)** and PAR4-AP **(K)** induced P-selectin release from washed platelets from mice (n = 5 independent experimental animals). **(L–O)** ATP release from washed platelets in mice was induced by thrombin **(L)**, U46619 **(M)**, PAR4-AP **(N)** and collagen **(O)** (n = 5 independent experimental animals). **(P–R)** Wash platelet spreading in mice induced by U46619 **(P)**, mean spreading area of individual platelets **(Q)**, and proportion of spreading platelets to total platelets in the field of view **(R)** (n = 3 independent experimental animals). Differences between groups were assessed by one-way ANOVA followed by Dunnett’s *post hoc* test. Statistics are presented as mean ± SEM. *P < 0.05, **P < 0.01 and ***P < 0.001.

To directly assess the *in vivo* state of platelet activation in this model, we analyzed the functional responsiveness of isolated platelets. We found that hypoproteinemic (ADR) mice exhibited a significantly heightened platelet activation profile. The results demonstrated that, compared with WT mice, ADR mice exhibited significantly elevated levels of platelet aggregation (Figures 6A–E), integrin αIIbβ3 activation (Figures 6F–H), P-selectin release (Figures 6I–K), ATP release (Figures 6L–O), platelet spreading (Figures 6P–R). This comprehensive hyper-reactivity suggested that platelets circulating in ADR mice existed in a primed or pre-activated state. Crucially, supplementation with human albumin (ADR + Alb group) restored these functional parameters to levels indistinguishable from WT controls. In addition to establishing a renal hypoproteinemia model, we utilized CCl_4_ to induce chemical liver injury and construct a hepatogenic hypoproteinemia model (Supplementary Figure S4A). However, this modeling process was time-consuming, and the use of corn oil as a solvent for CCl_4_ could potentially cause hepatic steatosis. Notably, platelet aggregation did not differ significantly among WT, Oil, and CCl_4_ groups (Supplementary Figures S4B–F).

### 3.7 Albumin supplementation normalized thrombotic function *in vivo* without inducing a bleeding diathesis

**FIGURE 7:**
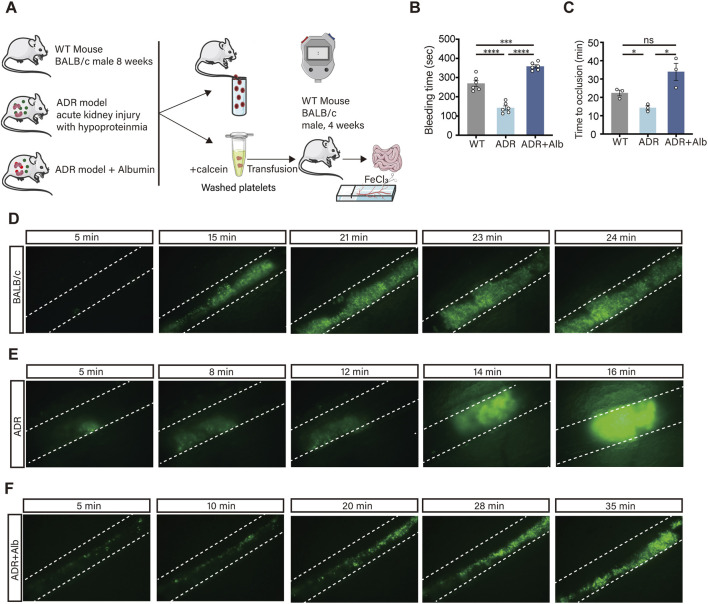
Albumin exerts an inhibitory effect on thrombus formation and hemostasis. **(A)** Flow chart of experiments on haemostatic function and thrombosis in mice. **(B)** Tail bleeding time in mice (n = 6 independent experimental animals). **(C–F)** Time of thrombosis induced in mouse mesenteric arteries using FeCl_3_ **(C)** (n = 3 independent experimental animals), WT **(D)**, ADR **(E)**, ADR Albumin **(F)** microscopic thrombosis in mice. Differences between groups were assessed by one-way ANOVA followed by Dunnett’s *post hoc* test. Statistics are presented as mean ± SEM. *P < 0.05, **P < 0.01 and ***P < 0.001.

The observed increase in platelet activation biomarkers,we tested this directly using two complementary *in vivo* assays (Figure 7A). The results indicated that the tail bleeding time in ADR mice was significantly shorter compared to WT mice, while the tail bleeding time in ADR Albumin mice was prolonged (Figure 7B). Platelets from these mice were isolated, labeled with calcein, and transfused into 4 weeks WT mice via the orbital venous plexus. A suitable mesenteric artery was selected and placed flat on a glass slide moistened with 0.9% saline. The artery was injured using filter paper soaked in 6% FeCl_3_, and thrombus formation at the injury site was observed. The findings revealed that, compared to WT mice, the time to platelet thrombosis in ADR mice was significantly reduced, whereas the thrombosis time in ADR Albumin mice was significantly prolonged (Figures 7C–F). Based on the aforementioned experimental results, using mouse models, we can conclude that: (1) Hypoproteinemia causes circulating platelets to enter an over - reactive state. This was verified by extracting single - cell suspensions of mouse platelets for comprehensive *in vitro* functional experiments. (2) Hypoproteinemia results in accelerated thrombosis *in vivo*. These results provided direct *in vivo* evidence that hypoproteinemia promoted a pro-thrombotic state characterized by elevated platelet activation biomarkers and enhanced thrombus formation, both of which were effectively counteracted by albumin replenishment.

## 4 Discussion

We were the first to use a series of eight albumin concentrations, ranging from 0 to the human physiological concentration of 40 mg/mL, in co-incubation with human washed platelets. Furthermore, for each *in vitro* platelet function assay, we used at least three different platelet stimulants. Our systematic approach revealed that albumin exerts a broad, concentration-dependent inhibitory effect on platelet activation, suppressing aggregation, integrin αIIbβ3 activation, P-selectin release, and ATP release. It also delayed platelet spreading and altered intracellular granule organization. These research findings acquired at clinically relevant albumin levels address the limitations of previous studies and emphasize the significant role of albumin as an endogenous platelet activity regulator.

Platelet activation, a critical process that initiates hemostatic responses and thrombosis, is triggered by the interaction between platelet receptors and their ligands (Cao et al., 2024). Key agonist receptors regulating platelet activation include Gq protein coupled, thromboxane A2, and collagen ITAM sequence, among others (Mack et al., 2024). These receptors modulate the production of second messengers. Subsequently, under the influence of kinases, these signals are integrated into various cellular responses, leading to platelet activation. Previous research has demonstrated that albumin can modulate platelet-activating receptor ligand responses and second messenger pathways through a range of indirect mechanisms. Therefore, we aimed to investigate whether albumin can directly modulate the phosphorylation of platelet-active kinase and play a direct role in influencing the platelet activation signaling pathway. Our data indicate that albumin concurrently inhibits the phosphorylation of both PKC and Akt, two central kinases in platelet activation. The observation that PKC activators (PMA and PDBu) fully restored platelet responses in the presence of albumin, whereas Akt activators (740Y-P and SC79) did not, which implied a hierarchical relationship within the inhibitory mechanism. It was hypothesized that PKC might be the primary and more direct target of albumin’s action. Albumin appeared to interfere with early signaling events upstream of PKC, such as phospholipase C activation or diacylglycerol (DAG) production. The inhibition of PKC phosphorylation would then lead to the secondary attenuation of Akt activation, given the known cross-talk between these pathways in platelets. This interpretation is consistent with the central role of PKC in integrin activation and granule secretion. A limitation of our current study is the absence of direct measurements of upstream signals like DAG or intracellular calcium flux, which would more accurately determine the initial site of albumin intervention. Future work employing such measurements will be essential to validate this proposed hierarchy and fully delineate the signaling sequence.

The inhibitory effect is intimately linked to albumin’s intrinsic biophysical property—its high density of surface negative charges. It is well established that albumin is a single-chain polypeptide consisting of 585 amino acid residues, with a molecular weight of 66,458 Da. The molecule contains 17 disulfide bonds and lacks glycosylation. At physiological pH 7.4, each albumin molecule can carry over 200 negative charges, making it the most negatively charged molecule in plasma (Richter-Bisson et al., 2024). Based on this structural characteristic, the charges on the surface of the albumin molecule were removed, and the retention of its secondary structure was confirmed via circular dichroism data. Nevertheless, neither was the albumin functional ligand binding experiment conducted, nor was the integrity of its tertiary structure and functional sites verified. These tasks need to be carried out in subsequent work. Mechanistically, it is proposed that the high negative charge density of albumin exerts its effect via electrostatic repulsion. This repulsive force may: (1) Impede albumin from approaching the negatively charged platelet membrane or specific membrane microdomains closely, thus disrupting receptor clustering or early signaling events; (2) Directly interfere with the fusion of negatively charged granule membranes (especially dense granules) with the plasma membrane during secretion, which accounts for the significant inhibition of ATP release and the altered intraplatelet granule distribution observed by transmission electron microscopy. This electrostatic barrier force of albumin plays a crucial role in inhibiting platelet spreading and degranulation.

In the investigation of the impact of albumin on platelet function, an interesting phenomenon was observed beyond the prevalent inhibitory conditions. When the specific agonist TRAP - 6 of PAR1 was employed, albumin inhibited ATP release but exerted no influence on aggregation or P-selectin release. This finding is considered to indicate the specificity of albumin’s action, and there may be certain mechanisms to account for this phenomenon. Firstly, in comparison to the binding of thrombin or ADP to PAR1, PAR4, or other G-protein-coupled receptors, the binding of TRAP - 6 to PAR1 differs in terms of sensitivity and the intensity of the signal cascade it generates, particularly in the mechanism of dense granule release. Secondly, there are substantial differences in the secretion kinetics of α-granules (which contain P-selectin) and dense granules (which contain ATP). The release of dense granules is a slower and more sustained process, which might be more prone to the inhibitory effect of albumin on granule-membrane fusion via charge mediation. Finally, the release of P-selectin is rapid and transient, and it cannot be compared with the slow and sustained overall release of ATP, and thus is less likely to reflect the continuous inhibitory effect. Therefore, it is believed that this observation highlights the specificity of albumin’s inhibitory effect and also demonstrates the complexity of platelet activation pathways. Future research findings regarding PAR1 signaling and granule release dynamics will further elucidate this mechanism.

Our *in vivo* studies using ADR model mice also confirmed that hypoproteinemia creates a prothrombotic environment. Washed platelets isolated from hypoproteinemic mice also showed an overreaction *in vitro* functionally, indicating that they were in a pre-activated state *in vivo*. This pre-activated state directly led to accelerated thrombosis in the injury model, and albumin supplementation could completely reverse this phenomenon. In the forthcoming experiments, we intend to incorporate the platelet factor 4 (PF4) and other plasma biomarker tests. These tests will be utilized as well - established, directly detectable platelet activation - specific biomarkers in the systemic circulation to further corroborate the evidence of an enhanced platelet activation status.

In recent years, numerous studies have confirmed that albumin can prevent thrombosis via multiple mechanisms, including the regulation of blood viscosity, inhibition of platelet aggregation, and stabilization of endothelial function (O'Donoghue et al., 2025; Yuan et al., 1995; Eligini et al., 2022; Mitra et al., 2025). Furthermore, albumin exhibits several crucial characteristics. For instance, it participates in oxidative stress via the Cys34 site and facilitates the efficient transport of endogenous and exogenous substances due to its biological affinity. These biological attributes collectively contribute to its function in antithrombosis (Luo et al., 2023; Vaishnav et al., 2024; Zhang et al., 2025). Our research provides a solid mechanistic basis for re-evaluating the role of albumin in the clinical control of thrombotic risk. In complex prothrombotic and inflammatory conditions, such as sepsis, major surgery or acute ischemic events (such as stroke), the accompanying hypoproteinemia may lead to serious consequences. Our research findings regarding the mechanism through which albumin directly inhibits platelet activation and thrombosis formation also offer a rational biological explanation for the correlation between hypoproteinemia and adverse cardiovascular outcomes. A recent large scale retrospective cohort study (involving the analysis of over 500,000 patients) revealed that ischemic stroke patients with reduced serum albumin levels at the time of admission had a significantly higher combined risk of early cardiovascular complications and death within 30 days compared to those with normal albumin levels. Notably, the risk of specific thrombotic events (such as myocardial infarction) was significantly increased in the hypoproteinemia group (Bucci et al., 2024). These findings indicate that serum albumin is not merely a nutritional indicator but also a crucial regulator of thrombotic risk after a stroke. Therefore, monitoring albumin levels can assist in risk identification, and our study further validates the rationality of this hypothesis. Whether albumin supplementation guided by specific thresholds can serve as an adjunctive treatment for high-risk hypoalbuminemic stroke patients to improve prognosis is also a hypothesis deserving of future prospective clinical evaluation.

There are also numerous innovative aspects concerning the clinical transformation and application of albumin. At present, the application of albumin is predominantly restricted to the intravenous injection of natural albumin (Liu et al., 2024; Zhang et al., 2025; Kim et al., 2025). In contrast, many new targeted anticoagulant drugs lack biological affinity, which makes drug delivery challenging and presents a risk of increased bleeding. Therefore, the biological characteristics and anticoagulant effects of albumin can be integrated with new targeted drugs, either as carrier molecules or as components of hemostatic adjuvants. Consequently, the future application of albumin in antithrombotic therapy still possesses significant potential.

## 5 Conclusion

**FIGURE 8:**
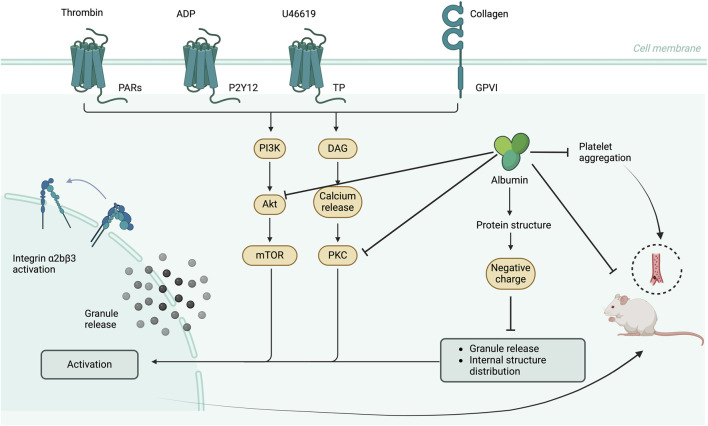
Schematic representation of the mechanism by which albumin inhibits platelet activity.

In conclusion, albumin effectively inhibits platelet thrombus formation without inducing bleeding risk. It suppresses agonist-induced washed platelet aggregation, membrane surface integrin activation, P-selectin release, and ATP release. Additionally, albumin prevents platelet spreading and alters the intraplatelet granules distribution. Furthermore, it inhibits the phosphorylation of kinases PKC and Akt within the platelet activation signaling pathway. The inhibitory effects of albumin on platelet activation are significantly attributed to its negatively charged surface (Figure 8).

## Data Availability

The original contributions presented in the study are included in the article/Supplementary Material, further inquiries can be directed to the corresponding authors.
