# Supplementary material for: Role of albumin in regulating platelet function
Source: Front Pharmacol. 2026 Feb 20;17:1734694. doi: 10.3389/fphar.2026.1734694 (PMC12962907; doi:10.3389/fphar.2026.1734694)
Supplement: Supplementary file 1 [file DataSheet1.docx]

Supplementary Material

## Supplementary figures

##
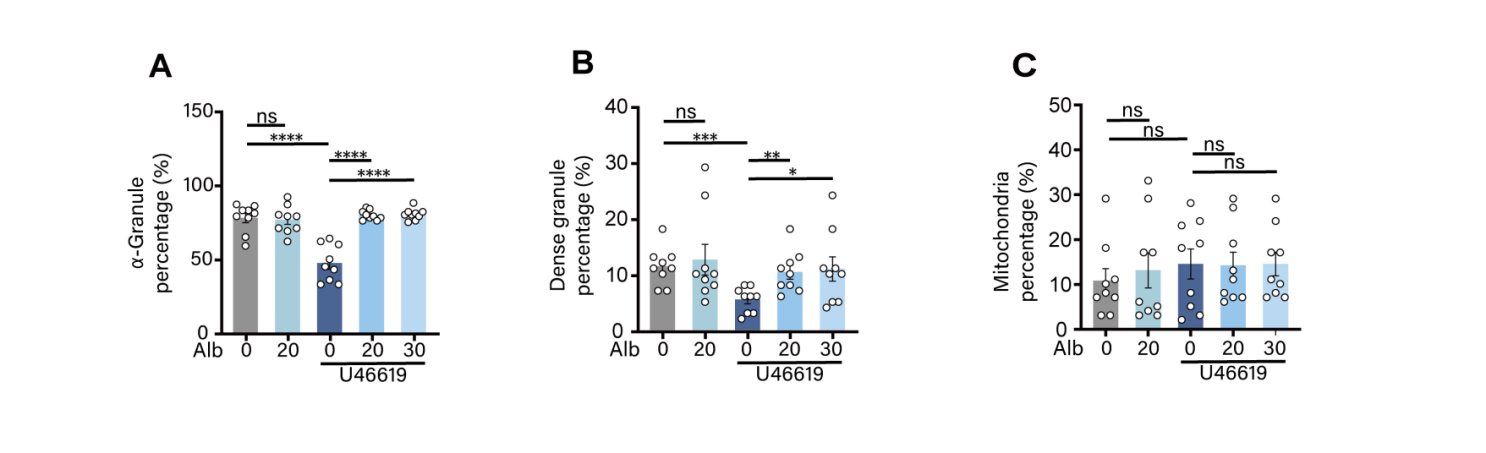
**Supplementary Figure 1.** Human plasma albumin inhibited granule release and affected internal granule distribution in human washed platelets. (A-C) Transmission electron microscopy field of view of platelets induced by agonist 200 nM U46619 at different concentrations of human plasma albumin (20, 30 mg/mL), relative counts of α-granules (A), dense granules (B), mitochondria (C) (n=3 independent experimental subjects, counted 3 microscope field of view per individual). Differences between groups were assessed by one-way ANOVA followed by Dunnett’s post hoc test. Statistics are presented as mean ± SEM. **P* < 0.05, ***P* < 0.01 and ****P* < 0.001.

##
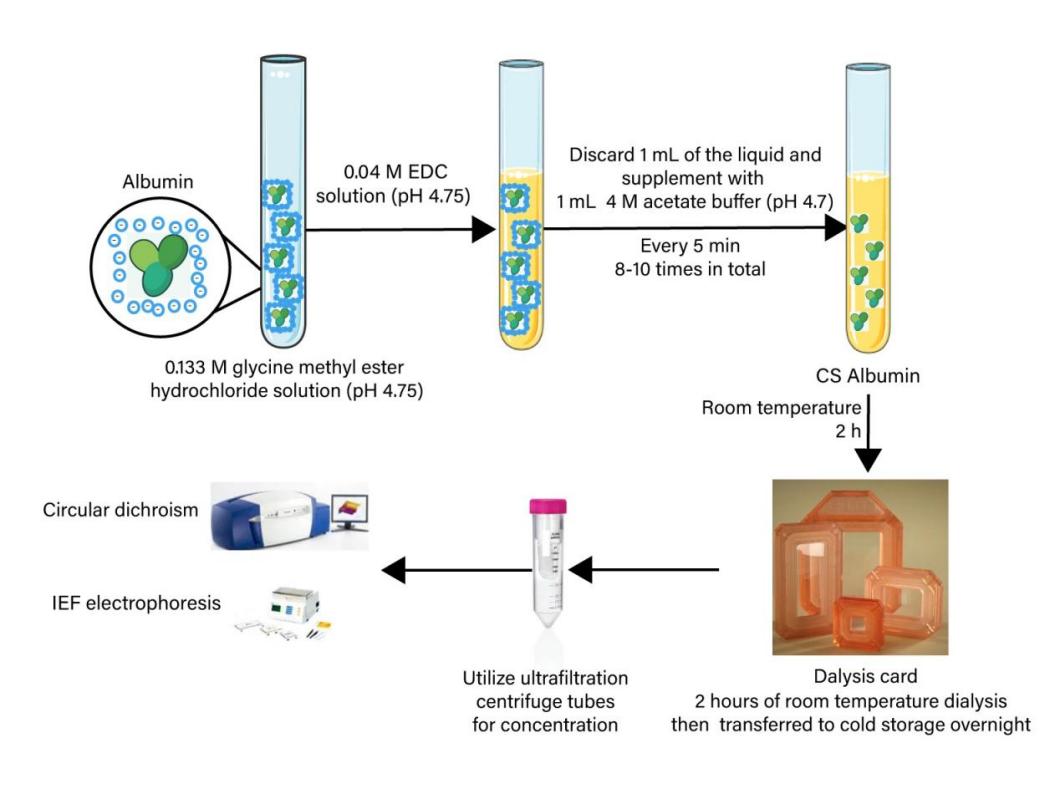


**Supplementary Figure 2.** Neutralization of the charge on the albumin surface.


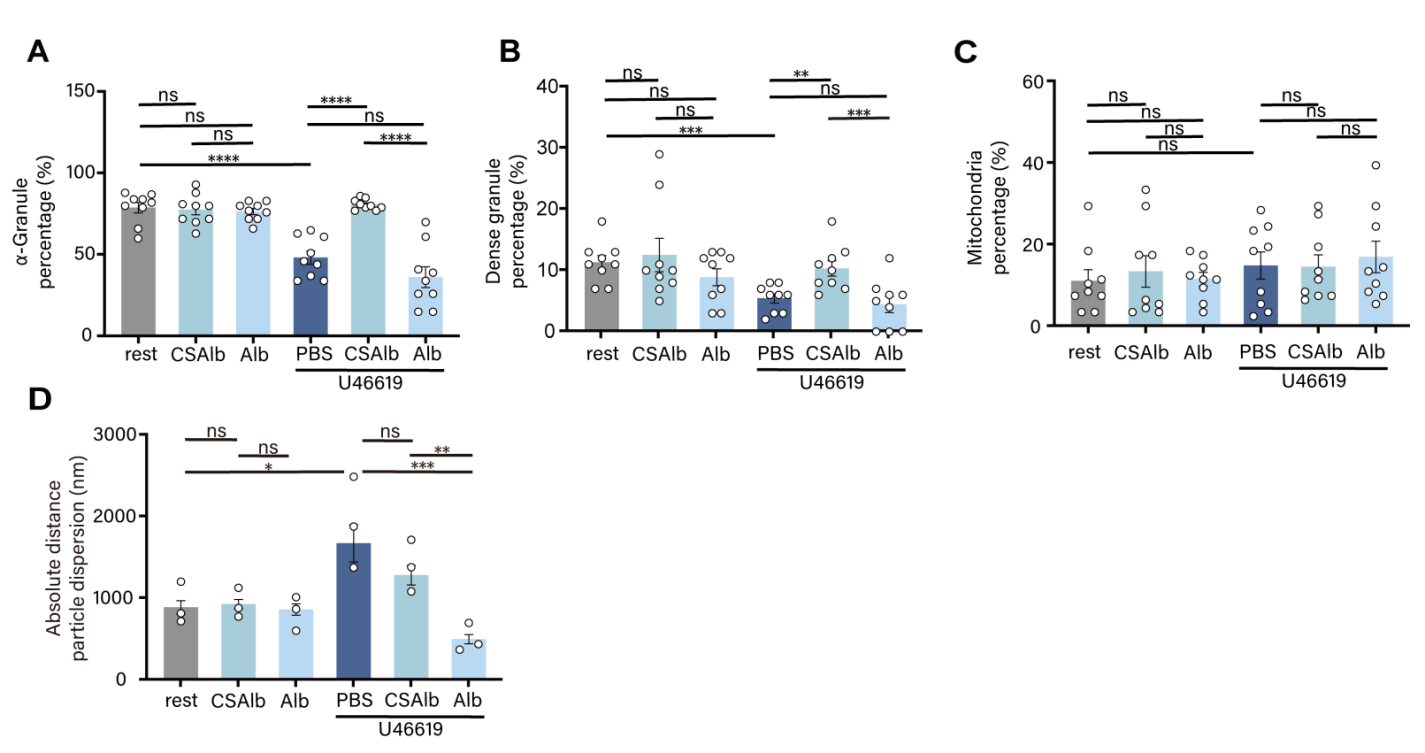


**Supplementary Figure 3.** Albumin influenced platelet aggregation, ATP release, granule release and internal granule arrangement via surface charge. (A-D) Transmission electron microscopy was used to observe the effect of CS albumin and natural albumin (20.0 mg/mL) on the internal granule arrangement in platelets before and after activation induced by 200 nM U46619. Effects of CS albumin and natural albumin (20.0 mg/mL) on the relative number of α-granules (A), dense granules (B), mitochondria (C) and the absolute distance of granules from the centre of the cell (D) in platelets (n=3 independent experimental subjects; A-C, counted 3 microscope field of view per individual; D, counted the distance from all granules to the center of the cell and calculated the average). Differences between groups were assessed by one-way ANOVA followed by Dunnett’s post hoc test. Statistics are presented as mean ± SEM. **P* < 0.05, ***P* < 0.01 and ****P* < 0.001.


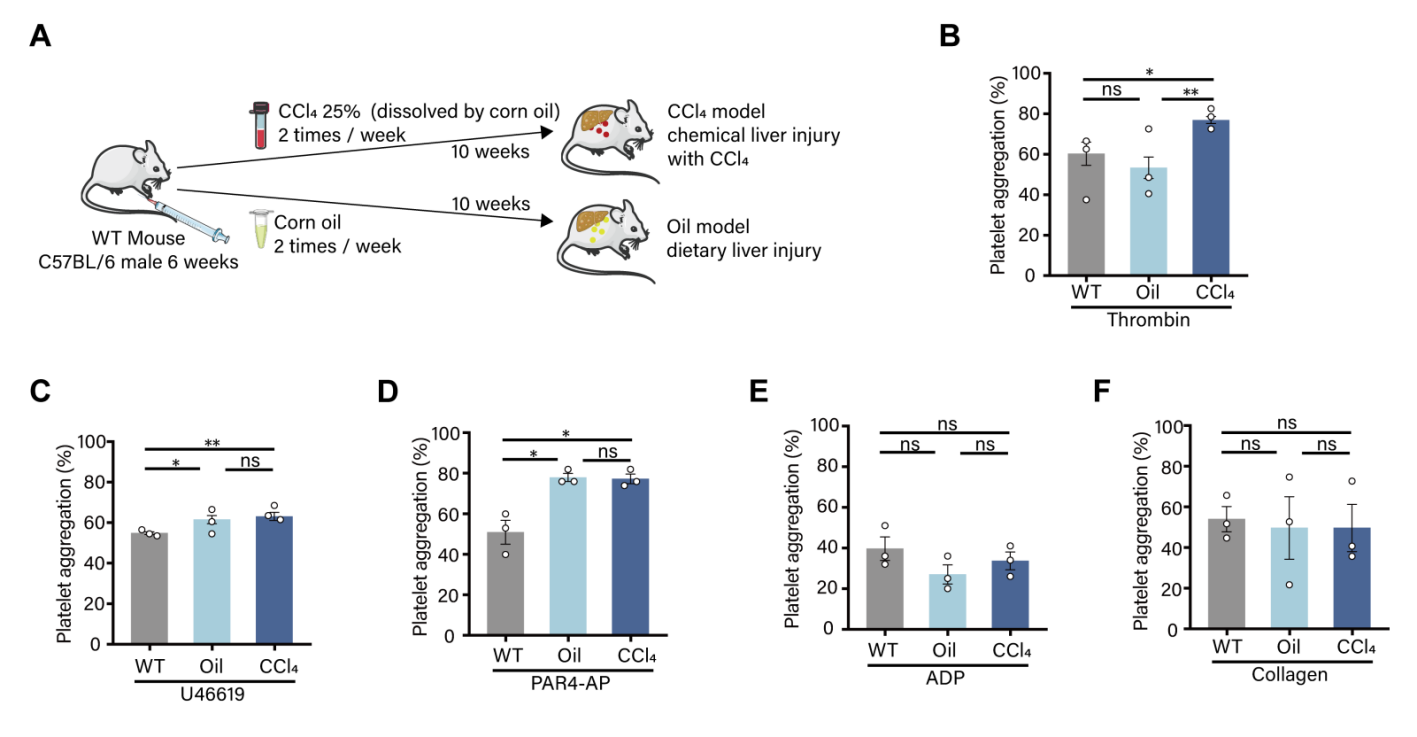


**Supplementary Figure 4.** Hypoproteinemia was found to promote platelet activation. (A) Utilized CCl_4_ to induce chemical liver injury and construct the hepatogenic hypoproteinemia model. (B-F) Wash platelet aggregation in WT, Oil and CCl_4_ mice induced by 0.01 U/mL thrombin (B), 200 nM U46619 (C), 40 μM PAR4-AP (D), 200 μM ADP (E), 1 μg/mL collagen (F) (n=5 independent experimental animals). Differences between groups were assessed by one-way ANOVA followed by Dunnett’s post hoc test. Statistics are presented as mean ± SEM. **P* < 0.05, ***P* < 0.01 and ****P* < 0.001.
